# Health Literate Internet-Based Information-Seeking Processes: Theory-Based Development of a Conceptual Model

**DOI:** 10.2196/39024

**Published:** 2023-03-23

**Authors:** Andrea Heiberger, Carolin Dresch, Anja Alexandra Schulz, Markus Antonius Wirtz

**Affiliations:** 1 Research Methods in Health Sciences Faculty of Mathematics, Natural Sciences and Technology University of Education Freiburg Freiburg im Breisgau Germany

**Keywords:** health literacy, internet-based search, model development, parents, health information, internet, childhood, allergy, prevention, pediatric, online, content, information

## Introduction

In a health care environment increasingly dominated by digital information, individuals must be able to identify valid digital health information, enabling them to communicate, make decisions, and act in a health-literate manner [1,2]. The generic health literacy (HL) model proposed by Sørensen et al [3] assumes four HL facets: *access*, *understand*, *appraise*, and *apply*. This model has to be adapted to validly characterize informational and decisional processes underlying targeted internet-based information-seeking (IBS) processes. This will provide the theoretical basis for upcoming analyses of empirical data on the IBS processes of parents for the health topic of early childhood allergy prevention (ECAP). ECAP is characterized by uncertainties regarding reliable evidence and recommendations [4].

## Methods

### Overview

The adaptation of the HL model followed a deductive literature-based approach. Considering models of cognitive psychology and health behavior, the focus was on characterizing information processing (IP) and search actions. After these model foundations had been concretized, specifically by topic and by individual experts (concept-mapping techniques [5]), the model structure was iteratively consented to in the public health expert group (N=10).

### Ethical Considerations

Ethics approval was not required for this deductive literature-based model development.

## Results

Health information seeking can be both problem and interest oriented [3]. Thus, individuals’ search competencies, their demands and needs for information (personal characteristics), and information characteristics (eg, availability) constitute components of the HL facet *access* [6,7]. Search results may serve to critically examine an individual’s prior knowledge, close knowledge gaps, or resolve uncertainties (deductive rather than systematic IP [6]). Alternatively, new facts or topics may be identified and explored (inductive rather than heuristic IP).

Information is processed in varying degrees of elaboration (*understand*) and is evaluated (*appraise*) in a knowledge- and action-oriented manner regarding the individual’s specific life situation. According to cognitive two-process models [8], IP may take place in an *automated* or habitual [6] manner. If problems arise or a deeper interest in information is stimulated, an increased cognitive effort with deepened *elaboration* is required (nonhabitual). Using the think-aloud technique in qualitative data analysis may be challenging because elaborated IP is more likely to be verbalized/recognizable than automated IP.

While semantic IP deals with content meaning, pragmatic IP concentrates on the adaptability to the individual’s life situation (cross-situational, depending on contextual factors of health or illness [9]).

*Appraisal* has proven to be crucial for integrating and using processed information. In the elaborated appraisal, besides comprehensibility, reliability, and trustworthiness (quality appraisal [10]), the meaning-related (semantic) and pragmatic aspects are assumed to be essential. For the latter, it makes a difference whether new behavior is considered (especially regarding individual relevance, threat, and risk perception) or already established behavior is critically reflected. Due to negative appraisal or demand for more in-depth information (immanent) or alternative contents (exmanent), a loop back to *access* may occur [9].

In IBS processes, appraisal is closely linked to *imaginated apply*, as individuals only consider a specific behavior if this seems realistically feasible. The decision to implement a behavior is influenced by self-efficacy expectations, control beliefs, and supporting and limiting conditions. Behavioral habits in the social environment and communicative exchange are also reflected. This results either in a behavioral intention or in a search for new information [6].

**Figure 1:**
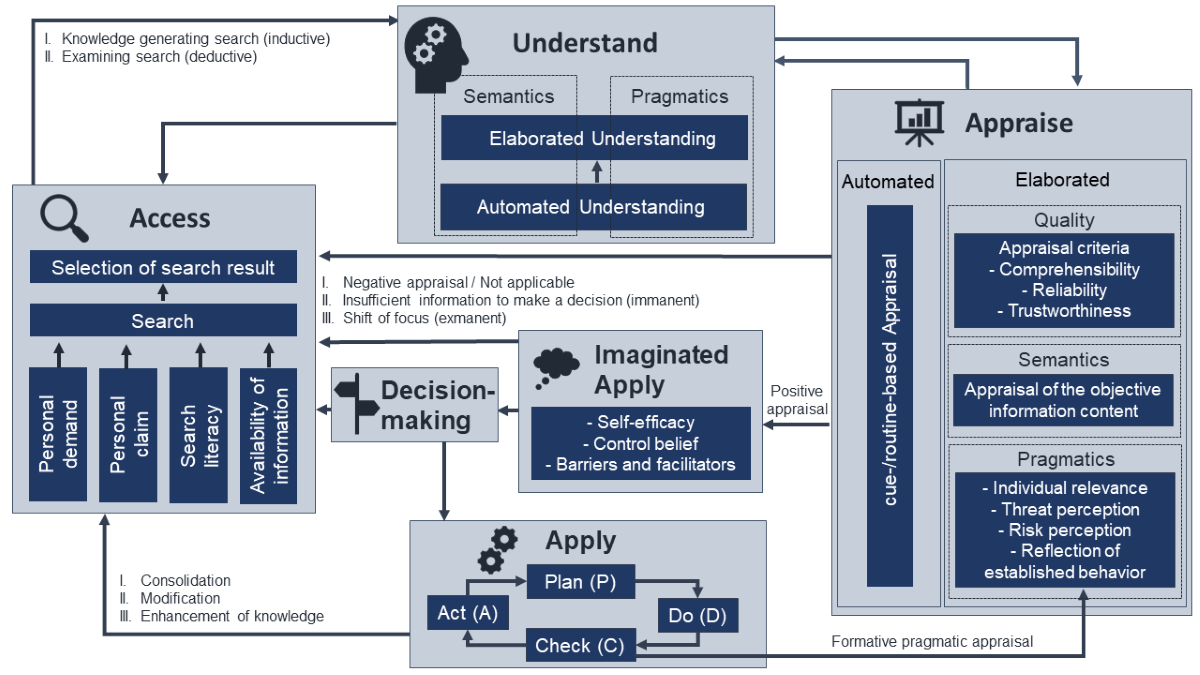
Adapted Health Literacy Model for IBS. IBS: internet-based information-seeking.

In general, feedback loops are characteristic of IBS processes. In typically fast and dynamic IP, clarifying and reassuring steps to the preceding *access*, *understand,* or *appraise* facets are distinct (Figure 1).

## Discussion

Existing HL models specify successive deliberate processes affecting HL-related decisions and behaviors in everyday life [2]. In IBS processes, decisions are mostly made automatically and spontaneously. It seems promising to combine the HL facets in Sørensen et al’s [3] model with risk information-seeking and processing models [6,7] to adequately account for the complexity and dynamics of the subprocesses underlying HL. The explicit distinction of person- versus information-related factors, semantic versus pragmatic IP, and actual versus imagined application may enhance the validity of HL models in general. Furthermore, it seems to be crucial to better understand in which conditions and to what extent IP occurs in an elaborated manner. The quality of available health information and processing-related cognitive load should be considered as moderating factors regarding all model aspects [9].

The suitability of the IBS process model as well as the necessity and completeness of all model elements will be validated using recordings of internet searches for information on the health topic of ECAP by new parents [4]. The content meaning of the model elements will be illustrated using qualitative empirical data for this specific health prevention topic.

## Abbreviations

ECAP: early childhood allergy prevention
HL: health literacy
IBS: internet-based information-seeking
IP: information processing
